# Supplementary material for: Time‑resolved multi-omic analysis of paclitaxel exposure in human iPSC‑derived sensory neurons unveils mechanisms of chemotherapy‑induced peripheral neuropathy
Source: Cell Death Dis. 2026 Feb 10;17(1):211. doi: 10.1038/s41419-026-08445-2 (PMC12921266; doi:10.1038/s41419-026-08445-2)
Supplement: Supplementary file 15 — Dataset 1. [file 41419_2026_8445_MOESM15_ESM.zip › CDD_Open_Data/04_RNA_Seq/Timeline_panel_plots_etc_2023-01-23_1609.zip!/Panel_Plots_etc/Evidence_Plots/72h/evidence_plot_standard contrasts_msigdb_hallmark_72h_PTX_vs_DMSO_ID0_M5890_HALLMARK_TNFA_SIGNALING_VIA_NFKB.pdf]

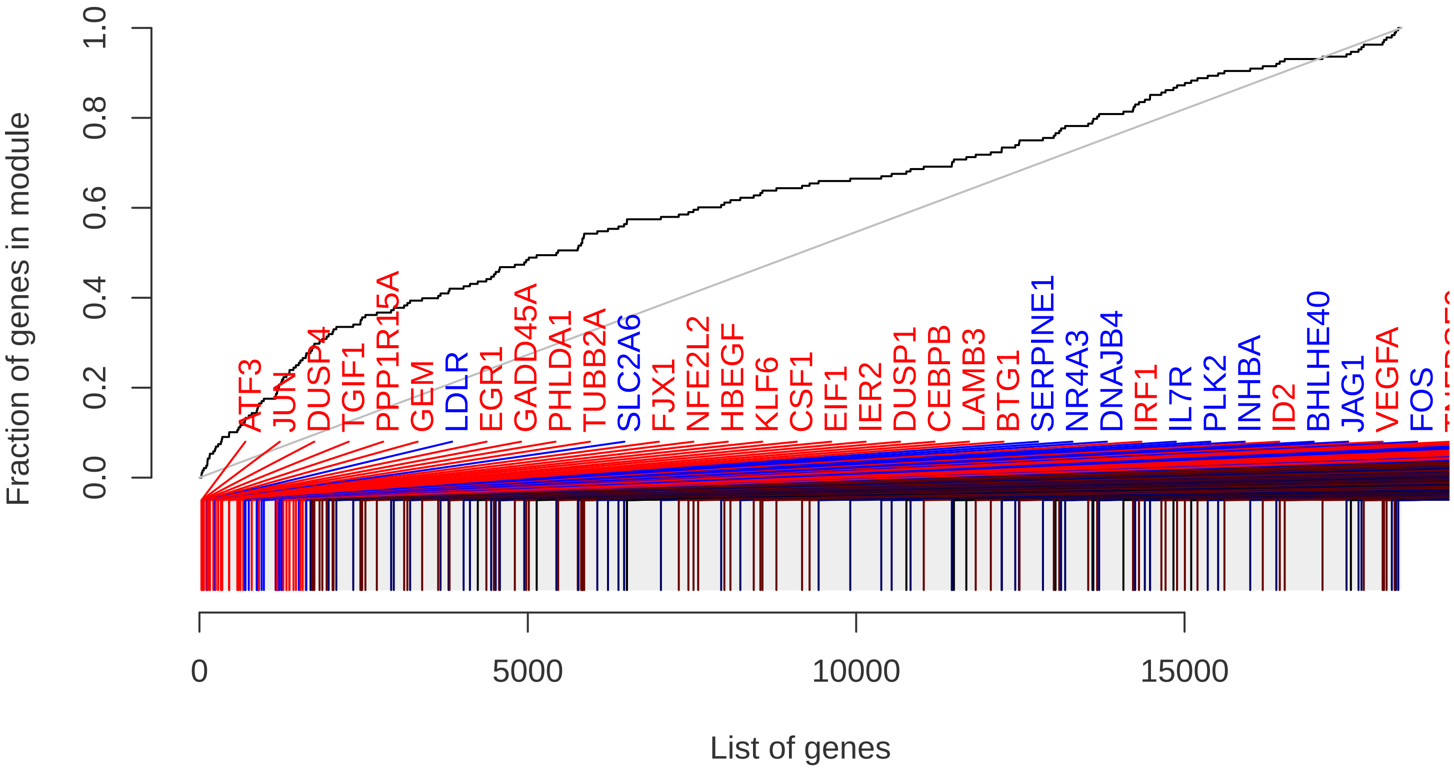

Fraction of genes in module
0.0
0.2
0.4
0.6
0.8
1.0
ATF3
JUN
DUSP4
TGIF1
PPP1R15A
GEM
LDLR
EGR1
GADD45A
PHLDA1
TUBB2A
SLC2A6
FJX1
NFE2L2
HBEGF
KLF6
CSF1
EIF1
IER2
DUSP1
CEBPB
LAMB3
BTG1
SERPINE1
NR4A3
DNAJB4
IRF1
IL7R
PLK2
INHBA
ID2
BHLHE40
JAG1
VEGFA
FOS
0
5000
10000
15000
List of genes
